# A Textbook of Clinical Anatomy

**Published:** 1904-04

**Authors:** 


A TEXTBOOK OF CLINICAL ANATOMY. For Students and Practitioners. By DANIEL N. EISENDRATH, A. B., M. D., Clinical Professor of Anatomy in the Medical Department of the University of Illinois (College of Physicians and Surgeons); Attending Surgeon to the Cook County Hospital, Chicago. Octavo, 515 pages, with 153 illustrations, a number in colors. Philadelphia, New York, London: W. B. Saunders & Company. 1903. (Cloth, $5.00 net; sheep or half morocco, $6.00 net.)

The subject of anatomy, and especially clinical anatomy, is so closely allied to practical medicine and surgery that it is absolutely impossible for a physician or surgeon to practise his profession successfully, unless he has an intimate knowledge of the human structure. The regular lectures in anatomy and the hours spent in the dissection room during the first year of a medical course, are far too short to give the student that thorough knowledge of the subject which is the cornerstone of medicine itself. The study of regional or topographic anatomy is rarely taught and, thorough as the student may be in the subject, he knows very little of applied anatomy or, as the author terms it, clinical anatomy.

The primary object of the work is to serve as a bridge for both practitioner and student from the descriptive anatomy as usually taught, to its daily application at the bedside or in the operating room. The author has gone to work with a thoroughness and precision that bespeaks much experience and a complete mastery of the subject in hand. He has mapped out the size and relations of the various organs,—the nerves, muscles, and bloodvessels to each other,—on the surface of the body, has made transverse sections showing the internal relation of organ to organ, the relation of muscles to bone in fractures of the latter, the resulting deformities, the relation of the epiphyses to the joint surfaces,—in short, he has covered the body with surface marking of every phase of anatomical development. Many of these markings are exceedingly clever and novel and are the results of painstaking study. The outlines have been marked upon a normal artist model then photographed, hence no error has been made in transferring.

The photographs have been reproduced in the highest style of art, and are printed on heavy glazed paper. The descriptions accompanying the illustrations are clear and concise and the rules for determining the various bony landmarks are of great assistance to the reader. The work, indeed, is superb,—text illustrations, paper, typography and binding being of unusual excellence. W. C. K.

